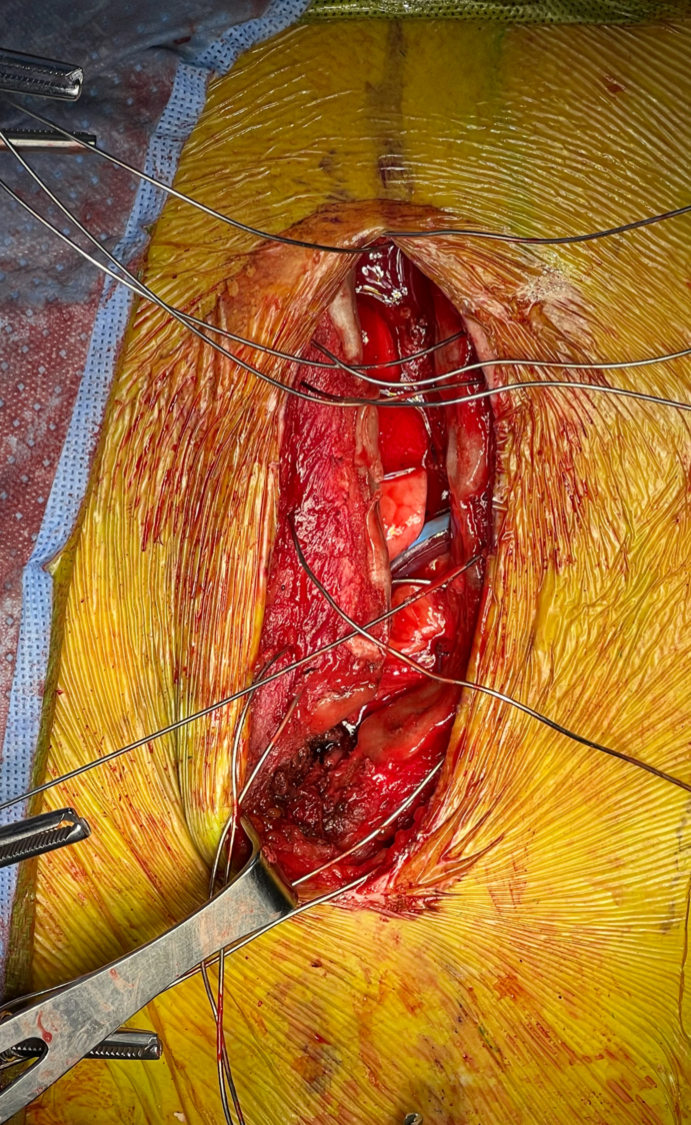# Minimally Invasive Pulmonary Thromboendarterectomy by a J-Shaped Upper Hemisternotomy

**DOI:** 10.1016/j.atssr.2025.05.017

**Published:** 2025-06-09

**Authors:** Yoshiya Toyoda, Mikiko Senzai, Akshay Chauhan, Sriram Vijayapuri, Suyog Mokashi, Roh Yanagida, Kewal Krishan, Hiromu Kehara

**Affiliations:** 1 Department of Cardiovascular Surgery, Lewis Katz School of Medicine, Temple University, Philadelphia, Pennsylvania

## Abstract

Chronic thromboembolic pulmonary hypertension is a rare but serious condition caused by pulmonary artery thrombi. Pulmonary thromboendarterectomy through median sternotomy is the “gold standard,” but minimally invasive approaches are emerging. We describe a 22-year-old female athlete with May-Thurner syndrome and factor V Leiden and prothrombin mutations in whom pulmonary embolism developed. After failed thrombectomy, she underwent pulmonary thromboendarterectomy by J-shaped upper hemisternotomy. Circulatory arrest time was 24 minutes; cardiopulmonary bypass time, 131 minutes; and cross-clamp time, 51 minutes. This technique is feasible and safe, achieving comparable outcomes to full sternotomy while improving cosmesis, making it an appealing option for selected patients.

Chronic thromboembolic pulmonary hypertension (CTEPH) is a rare but potentially fatal disease characterized by thrombi in the pulmonary arteries (PAs) leading to pulmonary hypertension.^1^ We perform about 60 pulmonary thromboendarterectomy (PTE) procedures annually for CTEPH and symptomatic chronic thromboembolic disease (CTED), gaining substantial experience in this complex surgery. Whereas PTE remains the “gold standard” treatment of CTEPH, minimally invasive approaches are continually sought to improve patient outcomes and cosmesis. This report describes the use of a J-shaped upper hemisternotomy approach.

A 22-year-old female collegiate soccer player with May-Thurner syndrome and family history of factor V Leiden mutation was also found to have heterozygous factor V Leiden mutation and heterozygous prothrombin gene mutation. She started oral contraceptives in April 2023 and experienced chest pain and dyspnea in September; bilateral pulmonary embolism with pulmonary infarct was diagnosed. Interventional radiology thrombectomy and thrombolysis were attempted but unsuccessful (Figure 1). A computed tomography scan revealed thrombus in the left common and internal iliac veins, and an inferior vena cava filter was placed. Despite anticoagulation, her symptoms improved but exertional dyspnea and tachycardia (heart rate up to 155 beats/min) persisted because of ventilatory insufficiency typical of symptomatic CTED. The left lower lobe segmental thrombus could not be retrieved, possibly leading to chronic embolus, and she was referred to our hospital (Figure 2).

**Figure 1:**
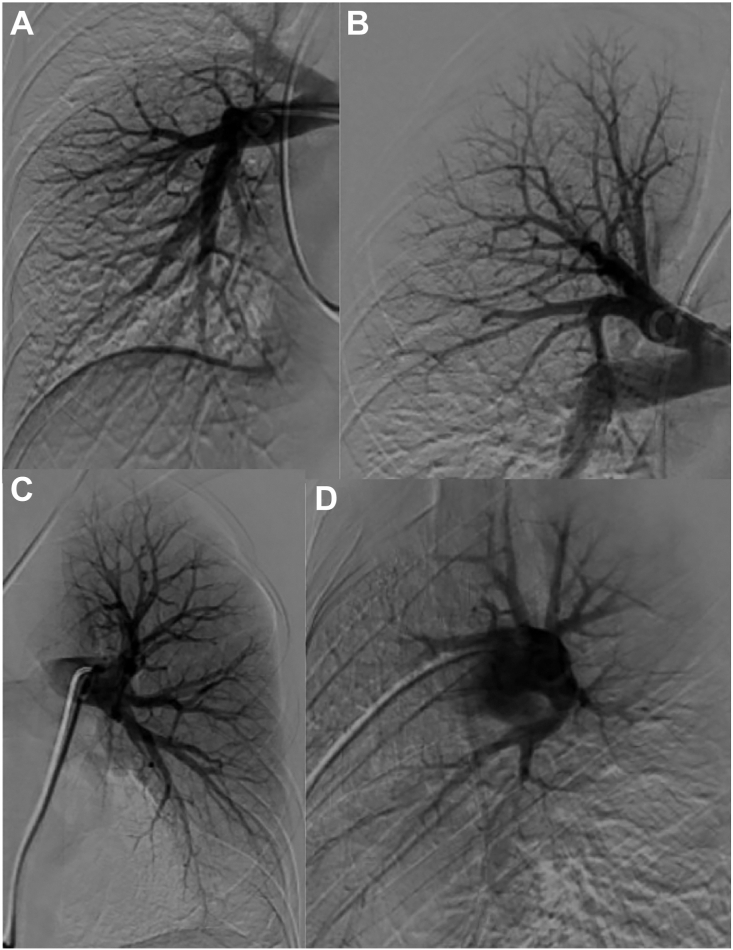
(a, b) No significant thrombus in the right pulmonary artery, with good perfusion of the right lung. (c) Good perfusion of left upper lobe and lingula. (d) No significant perfusion in the left lower lobe.

**Figure 2:**
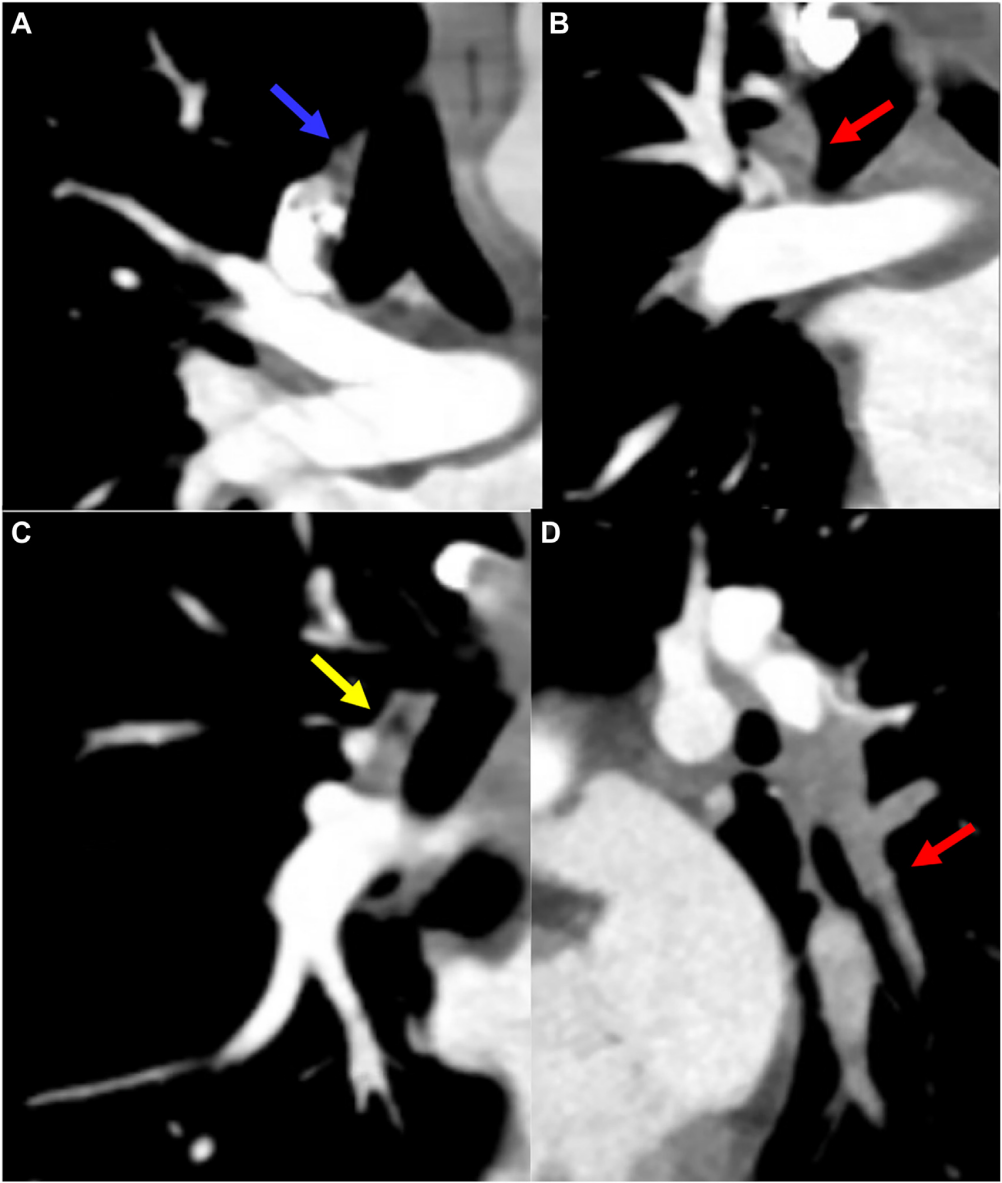
(a-c) Computed tomography angiography (axial view) showing increasing pulmonary emboli in the right lower segmental pulmonary arteries (PAs; blue arrow), extending into subsegmental PAs (red arrow), and new emboli in the right upper lobe PA (yellow arrow). (d) Filling defect in the left lower lobar PA, completely occluding the left lower lobe and lingular PAs (red arrow).

Preoperative cardiac catheterization revealed normal PA pressure of 22/5 (10) mm Hg, cardiac index of 2.28 L·min^−1^·m^−2^, and pulmonary vascular resistance of 0.79 Wood unit, with severely elevated systemic vascular resistance (20.93 Wood units). Perfusion scans showed normal right lung perfusion and a proximal clot in the left lung with reduced perfusion to the lower lobe and interlobar artery. Transthoracic echocardiography showed normal left ventricular ejection fraction (50%-55%) and normal right ventricular size and function. Because of persistent symptoms, the risk of CTEPH progression, and the patient’s young age and cosmetic concerns with a body mass index of around 20 kg/m^2^, we selected a minimally invasive approach.

Under general anesthesia in the supine position, a J-shaped upper hemisternotomy was performed to the right fourth intercostal space, preserving the right internal mammary artery and vein. After heparin administration, cardiopulmonary bypass (CPB) was initiated with arterial and venous cannulas for the aorta and superior and inferior venae cavae. Rapid cooling was initiated, maintaining an arterial-venous temperature gradient of ≤10 °C (Figure 3).

**Figure 3:**
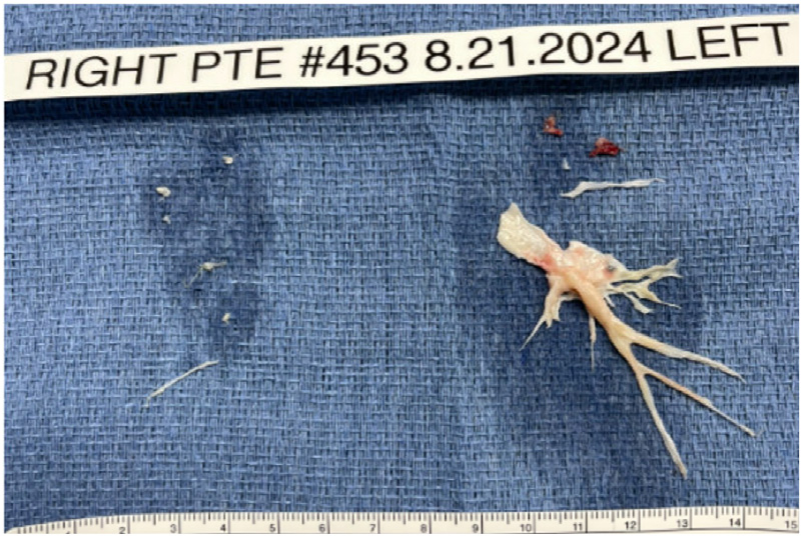
No clots were present in the right main, lobar, or segmental pulmonary arteries (type IV disease). A large clot was removed from the left lower lobe and a minimal clot from the left upper lobe (type II disease).

The main PA was looped, and the left PA was opened longitudinally, revealing left lower lobar clots with type II disease. The right PA was opened, showing no clots in the main, lobar, or segmental PAs, with type IV disease. After cross-clamping and cardioplegia, PTE was performed on the right PA for 9 minutes under circulatory arrest, removing a bridging clot from a subsegmental branch of the right lower lobe (Supplemental Figure 1). After 10 minutes of reperfusion, PTE for the left PA was completed in 15 minutes, removing a large clot from the left lower lobe and a minimal clot from the left upper lobe. The patient was weaned off CPB without issues after rewarming, and the procedure was completed. CPB time was 131 minutes, cross-clamp time was 51 minutes, and total circulatory arrest time was 24 minutes. Chest closure was uneventful (Supplemental Figure 2). The patient had an uneventful postoperative course and was discharged on postoperative day 5.

## Comment

Minimally invasive approaches for PTE, such as the inverted-T upper hemisternotomy^2^ and bilateral mini–anterior thoracotomies without cross-clamping,^3^ have been reported. However, these methods are not widely utilized.

In CTEPH, fibrotic transformation of PA clots and secondary microvasculopathy lead to increased pulmonary vascular resistance.^1^ Patients with CTED, defined by chronic thromboembolic obstruction without pulmonary hypertension (mean PA pressure ≤25 mm Hg),^4^ may benefit from PTE. However, evidence on the impact of PTE in CTED patients remains limited.^5^ Successful PTE has been reported in a small number of CTED patients without mortality,^6^ and early treatment with PTE can improve symptoms and exercise tolerance and potentially reduce long-term complications like small-vessel vasculopathy and right-sided heart failure.^3^ In this case, the patient was a young, active athlete and had failed to respond to both catheter-based interventions and medical therapy, making PTE a reasonable option.

De Vos and coworkers^2^ reported average CPB time of 274 minutes, cross-clamp time of 131 minutes, and circulatory arrest time of 56 minutes for PTE for CTEPH using an inverted-T upper hemisternotomy. Conventional sternotomy approaches reported CPB times of 248 minutes and cardiac arrest times of 138 minutes^7^; another study reported 231 minutes of CPB, 96 minutes of cross-clamp time, and 35 minutes of cardiac arrest.^8^ In this case, despite right-sided disease being classified as type IV, the J-shaped upper hemisternotomy approach did not have a negative impact on the surgical course based on CPB time, cardiac arrest time, and duration of arrest. This approach is a favorable option for young patients or those concerned with cosmesis.

This case highlights the use of a J-shaped upper hemisternotomy approach for PTE, which has been infrequently described. Although it is potentially less suitable for obese patients, it offers a simpler closure than inverted-T hemisternotomy or bilateral minithoracotomy.

PTE by a J-shaped upper hemisternotomy approach is feasible and safe, with outcomes comparable to full sternotomy, without prolonging CPB or circulatory arrest times. It allows bilateral treatment through a single incision, with few contraindications, making it a favorable option for cosmesis-conscious patients. Further studies are needed to assess long-term outcomes and to identify populations of patients who may benefit most from this technique.

## Figures and Tables

**Supplementary Figure 1:**
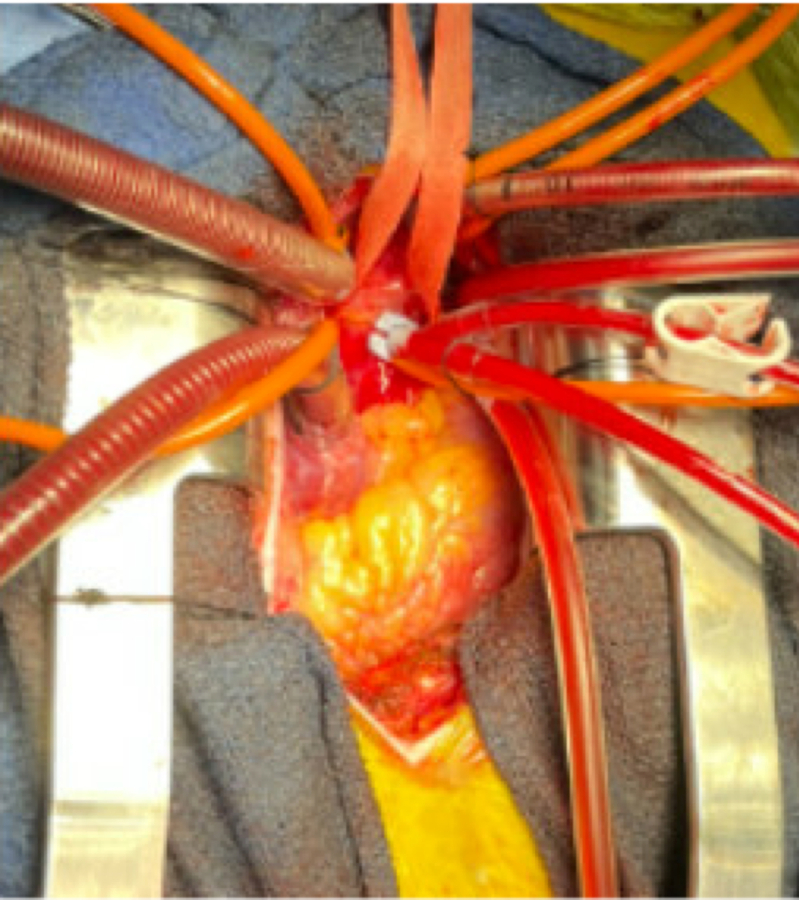


**Supplementary Figure 2:**