# Supplementary material for: Minimally Invasive Pulmonary Thromboendarterectomy by a J-Shaped Upper Hemisternotomy
Source: Ann Thorac Surg Short Rep. 2025 Jun 9;3(4):893–6. doi: 10.1016/j.atssr.2025.05.017 (PMC12712136; doi:10.1016/j.atssr.2025.05.017)
Supplement: Supplementary Figure Legends [file mmc1.docx]

**Supplemental Figure 1**

After heparin administration, CPB is established by dissecting and taping the aorta, superior and inferior venacave. Rapid systemic cooling is initiated, maintaining an arterial-to-venous temperature gradient within 10°C.

**Supplemental Figure 2**

Six pairs of sternal wires used for sternal closure.
